# Reviving the genitive. Prescription and practice in the Netherlands (1770–1840)

**DOI:** 10.1515/jhsl-2019-0016

**Published:** 2020-11-23

**Authors:** Andreas Krogull, Gijsbert Rutten

**Affiliations:** Leiden University Centre for Linguistics, Leiden University, Leiden, Netherlands

**Keywords:** Dutch, genitive case, historical sociolinguistics, language planning, prescriptivism

## Abstract

Historical metalinguistic discourse is known to often prescribe linguistic variants that are not very frequent in actual language use, and to proscribe frequent variants. Infrequent variants that are promoted through prescription can be innovations, but they can also be conservative forms that have already largely vanished from the spoken language and are now also disappearing in writing. An extreme case in point is the genitive case in Dutch. This has been in decline in usage from at least the thirteenth century onwards, gradually giving way to analytical alternatives such as prepositional phrases. In the grammatical tradition, however, a preference for the genitive case was maintained for centuries. When ‘standard’ Dutch is officially codified in 1805 in the context of a national language policy, the genitive case is again strongly preferred, still aiming to ‘revive’ the synthetic forms. The striking discrepancy between metalinguistic discourse on the one hand, and developments in language use on the other, make the genitive case in Dutch an interesting case for historical sociolinguistics. In this paper, we tackle various issues raised by the research literature, such as the importance of genre differences as well as variation within particular genres, through a detailed corpus-based analysis of the influence of prescription on language practices in eighteenth- and nineteenth-century Dutch.

## 1 Introduction

Historical metalinguistic discourse is known to often prescribe linguistic variants that are not very frequent in actual language use, and to proscribe frequent variants. An example of the former is the proposal made by the seventeenth-century German grammarian Schottelius to adopt <kk> instead of <ck> in words such as *wecken* ‘to wake’ (McLelland 2014: 266–267). An example of the latter is preposition stranding as in *who are you talking to*, which was quite common in sixteenth- and seventeenth-century English, and fiercely attacked by some eighteenth-century grammarians (Yáñez-Bouza 2015). Infrequent variants that are promoted through prescription can be innovations, as in the case of German <kk>. They can also be conservative forms that are on the way out, that have already largely vanished from the spoken language and are now also disappearing in writing. An extreme case in point is the genitive case in Dutch. This has been in decline in usage from at least the thirteenth century onwards, gradually giving way to analytical alternatives such as prepositional phrases. However, when the grammatical tradition emerged in the sixteenth century, it strongly prescribed the use of the genitive case. This preference for the genitive case was maintained for centuries. The striking discrepancy between prescription on the one hand and developments in language use on the other make the genitive case in Dutch an interesting case for historical sociolinguistics, and in particular for an analysis of the effectiveness of prescription on language practices.

The Dutch genitive case in general as well as the discrepancy between prescription and practice has attracted quite some attention in the research literature. Scott (2014) argues that prescriptive grammars were influential in preserving the declining genitive case in seventeenth- and eighteenth-century Dutch. Weerman et al. (2013) state that formal genres tend to preserve the genitive in seventeenth-century texts. Analysing private letters from the seventeenth and eighteenth century, respectively, Nobels and Rutten (2014) and Simons and Rutten (2014) claim that the genitive is particularly likely to be preserved in formulaic language. Furthermore, the analytical prepositional alternatives to the historical genitive case are increasingly acknowledged and accepted in eighteenth-century metalinguistic discourse (Rutten 2012). However, when ‘standard’ Dutch was officially codified in 1805 in the context of a national language policy (see Section 2 for an outline), the genitive case is again strongly preferred over the analytical alternatives (Rutten 2016a). As late as the early nineteenth century, therefore, metalinguistic discourse still aimed to ‘revive’ the synthetic genitive case. In this paper, we tackle the issues raised by the research literature, such as the importance of genre-specific differences as well as variation within particular genres, through a detailed corpus-based analysis of the influence of the official 1805 prescription on actual language use.

The research reported here is part of a growing body of historical-sociolinguistic literature on the interplay of language norms and language use. Recent publications such as Rutten et al. (2014) and Anderwald (2016) are generally sceptical with respect to the influence of prescriptive activities on patterns of variation and change, though some examples of prescriptive influence can certainly be found. Poplack et al. (2015) in their study of historical French state that language use is often much more predictable and diachronically less variable than the grammatical tradition, which rules out the possibility of normative influence. With respect to the official Dutch language policy of the early nineteenth century, Krogull et al. (2017) argue that the 1805 grammar of Dutch did not exert a strong influence on the use of relative pronouns in a corpus of historical Dutch of the time. Krogull (2018a, 2018b) and Rutten et al. (2020), however, show that a strong influence of the official norms can be assumed for various orthographic variables. This may mean that there is a difference between (not so effective) grammatical and (possibly highly influential) orthographic prescriptions, but we hypothesise that the level of social awareness may interfere: contrary to the relativisers discussed by Krogull et al. (2017), the genitive case has been a core topic in the Dutch grammatical tradition from the sixteenth century onwards.

The Dutch situation in the decades around 1800 offers a perfect test case for establishing possible influences of prescription on language practices. As mentioned above, an official top-down language policy was developed, resulting in the official codification of Dutch in 1804/1805. This was a novelty as there had been no official interference with language up to then. Against the background of the newly developed standard language ideology, which came into existence as the linguistic counterpart of the wider phenomenon of cultural nationalism (Rutten 2016b), policy measures aimed to regulate language use in the educational and administrative domains. The Dutch case therefore also allows us to empirically assess the significance of implementation and acceptance in the sense of Haugen (1966, 1987), which have not attracted the amount of historical-sociolinguistic attention they deserve (cf. Rutten et al. 2020).

In section 2, we describe the historical-sociolinguistic context of the present research, focusing on eighteenth-century nationalism, and the official language planning efforts it gave rise to in the Netherlands. Section 3 discusses the genitive case and other genitival constructions in the history of Dutch, concentrating on the linguistic changes over time as well as on the metalinguistic discourse accompanying these changes. In section 4, we introduce our historical corpus and how we used it for this study. Section 5 comprises the results, followed by the discussion in section 6.

## 2 Nationalism and language policy

The research presented here is part of a larger research project that focuses on a sociolinguistic event of crucial importance to the history of Dutch.1The project is called *Going Dutch. The Construction of Dutch in Policy, Practice and Discourse, 1750–1850* (VIDI grant awarded to Gijsbert Rutten by the Netherlands Organisation for Scientific Research (NWO), 2013–2018). Project members are the present authors, Bob Schoemaker and Marijke van der Wal. In the course of the eighteenth century, cultural nationalism developed in the Low Countries, and in fact in large parts of Europe (cf. Leerssen 2018a). Within a few decades, this resulted in an official top-down language policy in the northern parts of the Low Countries, i.e. in the Netherlands. A key aspect of most manifestations of cultural nationalism in Europe is a strong focus on the intimate relationship of *language* and *nation*. Nationalist thinking can be discerned in many cultural fields, including literature, the arts, history-writing, music and so on, but, as Leerssen (2018b: 23) comments, foremost among these “is clearly that of language. From Herder to the generation of the Humboldts, Schlegels and Grimms, language comes to be seen as the essential soul of a nation’s identity and position in the world. An extraordinary number of cultural-nationalist initiatives are concerned with language: from grammar-writing to purism, from language revivalism to language planning”. The close connection between language and nation also characterises the Low Countries in the late eighteenth and early nineteenth century. Language played an increasingly important part in cultural-nationalist discourses in the second half of the eighteenth century (Rutten 2016b). In tandem with the equally increasing call for linguistic uniformity, this period saw the emergence of modern standard language ideology (Milroy and Milroy 2012; Lippi-Green 2012; Rutten 2016b, 2019). In line with the strong educational focus of the Dutch Enlightenment, emphasis was also placed on the need for a national school system, which would ensure the transmission of the national language to younger generations (Rutten 2019; Schoemaker 2018). Thus, Dutch pedagogical discourse of the late eighteenth century brought various elements together: the need to ‘enlighten’ the people, the conviction that education was the main social domain in which such enlightenment should take place, and the belief that a uniform language was an instrument of crucial importance in this. This instrument would bind the nation together, revealing its historical essence, as well as enable the members of the nation to successfully communicate with each other in a newly established political constellation. As such, Dutch nationalism of the times combines the Romantic-nationalist model typically associated with the German tradition, and the civic-nationalist model characteristic of the French situation (Wright 2012).

Characteristic of the Dutch case is the almost immediate implementation of these newly developed cultural-nationalist ideas in concrete policy measures such as educational reforms (Schoemaker 2018). In the first decade of the nineteenth century, several school acts (1801, 1803, 1806) were passed that sought to bring the complex educational system under national control, as until then, many local, regional and church authorities had their own educational policies throughout the Low Countries. A national school inspection system was established in 1801. A national list of officially approved schoolbooks was announced. Importantly, the grammar of Dutch became a mandatory subject in public primary education. In the wake of these changes, teacher-training institutes were established. Many school inspectors and schoolteachers were also authors of schoolbooks. School inspectors were responsible for teacher examinations, and they usually also promoted teacher societies in their districts. Within these societies, teachers from one or more school districts would come together to discuss educational and pedagogical matters, including matters related to the Dutch language such as its grammar and spelling.

Whereas grammar played a marginal role in eighteenth-century primary education, the strong focus on language in the new school system from c. 1800 onwards led to the publication of many new grammar books for the use in schools. Most of these new schoolbooks were founded on the orthography written by Siegenbeek (1804) and the grammar devised by Weiland (1805), both of which were commissioned by the Minister of National Education. In fact, these two publications were the linguistic heart of the new language and language-in-education policies of the government. Together, they were the concrete outcome of what was called the *schrijftaalregeling* ‘written language regulation’. The *schrijftaalregeling* was the governmental effort to create a uniform Dutch language, primarily in writing, which was to be used in the educational and administrative domains. Thus, Siegenbeek’s spelling proposal and Weiland’s grammar constitute the first official codification of the Dutch language. On c. 400 pages in octavo, Siegenbeek (1804) comprises an elaborate discussion of spelling principles, overviews of historical orthographic practices, analyses of disputed features as well as an alphabetical word list. Weiland’s (1805) grammar gives a short orthographic overview, referring the reader to Siegenbeek, while devoting most of its c. 350 pages in octavo to morphology and syntax. Together, Siegenbeek (1804) and Weiland (1805) constitute the officially codified version of Dutch. It is this official codex that was considered to be the only true Dutch language and it was an important task of school inspectors to make sure that schoolteachers throughout the country taught this variety to schoolchildren – often with the explicit effort to simultaneously eradicate other, particularly local varieties (Schoemaker and Rutten 2017).

In sum, the decades around 1800 in the Low Countries witnessed a strong nationalisation of many social and cultural domains, including language and education, resulting i.a. in a top-down intervention aimed at the homogenisation of a highly variable linguistic situation (see e.g. Rutten and van der Wal 2014). Elsewhere, we have focused on the ideological embedding of this intervention, situating it in the context of the rise of standard language ideology and cultural and political nationalism generally (Rutten 2016b), on the implementation of the new policies in the educational domain (Schoemaker and Rutten 2017), and on the diffusion of some of the newly codified language norms in language use (Krogull et al. 2017; Krogull 2018a). We will take up the latter theme in this paper through an analysis of the genitive case in historical Dutch.

## 3 The genitive case in Dutch

As a West Germanic language, historical stages of Dutch displayed a fully-fledged case system. More specifically, the oldest Dutch sources, dating back to the ninth to eleventh centuries, provide evidence that the Dutch language comprised a nominative, genitive, dative and accusative case, resulting in inflection on pronouns, adjectives and nouns, which moreover followed the historical three-way gender system (masculine, feminine, neuter). As in most West and North Germanic languages, both case and gender have vanished to a considerable extent in more recent stages of Dutch. At present, remnants of the old case distinctions are mostly found in personal pronouns, where particularly the nominative-accusative distinction has been maintained. Analytical constructions with prepositions have increased throughout the centuries, gradually taking over functions previously fulfilled by case. The three-gender system has survived in southern parts of the language area, but most varieties of Dutch have shifted to a two-gender system, conflating the masculine and the feminine gender into one category.2The current situation and the history of the gender changes are arguably more complex than summarised here, see e.g. Audring (2006) for an analysis of gender in present-day Dutch.


In the oldest Dutch sources, evidence of case syncretism can already be found. The genitive and dative singular of feminine nouns and demonstrative pronouns, for instance, are identical, as are the dative and the accusative of most personal pronouns (Quak and van der Horst 2002: 40, 43, 44). Furthermore, analytical constructions such as prepositional phrases also already occur in the oldest sources. This means that we are dealing with changes with a considerable time-depth, and with extremely long S-curves spanning more than one thousand years. Nonetheless, some decisive stages can be discerned in the textual evidence. For example, the strong focus on grammatical gender in metalinguistic discourse from the late sixteenth century onwards clearly suggests that the masculine-feminine distinction was already declining, or was in fact already nonexistent to quite a few language users (e.g. Geerts 1966; van den Toorn et al. 1997: 298–300).

Table 1 gives an idealised overview of the changes in genitival constructions in the history of Dutch, focusing on the definite article (van Loey 1980: 43–44; Quak and van der Horst 2002: 44–45; van der Wal and van Bree 2008: 135). Obviously, more variation can be found in the sources, including other types of case marking and different prepositions, and the periodisation is nothing other than a helpful simplification. Nonetheless, Table 1 shows the shift from synthetic genitives in the oldest sources to periphrastic alternatives in the more recent sources. In the Old Dutch period, synthetic forms predominated. From Early Modern Dutch onwards, analytical forms have been dominant. Note that the definite article developed from the demonstrative pronoun. Vowel reduction can be seen in the change from <ie> to <e> in the masculine and feminine singular and in the plural, and from <a> to <e> in the neuter singular, where the <e>-spellings allow for a pronunciation as schwa. At present, genitive case marking survives to some extent only in specific contexts (Scott 2014).

**Table 1:** Idealised overview of genitival constructions in the history of Dutch.

	Old Dutch until c. 1150	Middle Dutch c. 1150–1550	
	*demonstrative pronoun*	*definite article*	*preposition + definite article*
	*synthetic*	*synthetic*	*analytical*
*masc sing*	thes	dies/des	van dien
*fem sing*	thero	dier/der	van die
*neut sing*	thes	dies/des	van dat
*plural*	thero	dier/der	van die
Early and Late Modern Dutch c. 1550–1900			Present-day Dutch
	*definite article*	*preposition + definite article*	*preposition + definite article*
	*synthetic*	*analytical*	*analytical*
*masc sing*	des	van den	van de
*fem sing*	der	van de	van de
*neut sing*	des	van het	van het
*plural*	der	van de	van de

Metalinguistic discourse has been promoting four to six nominal cases from the sixteenth century onwards. In addition to the aforementioned four cases, language commentators often distinguished an ablative and a vocative case, following the descriptive model of traditional Greek, and in particular Latin grammar (Dibbets 1995: 159–165). In other words, Early Modern Dutch metalinguistic discourse was conservative, and moreover archaic grammatical categories, known from the classical languages, were often ascribed to modern Dutch. In just about every Early Modern Dutch grammar, the more common four nominal cases were considered vital parts of Dutch morphology, constituting the central part of the morphology section alongside verbal inflection. At the same time, it is usually assumed that particularly the genitive case, which had been declining since the Middle Ages, was hardly used in spoken Dutch of the time. Scott (2014: 107) states that the analytical construction with the preposition *van* ‘of’ “had become constructionalised as an alternative to the adnominal genitive” by the Middle Dutch period. According to Weerman and de Wit (1999), the turning point for the Dutch genitive should be located in the thirteenth and fourteenth centuries. In the 1300s, synthetic genitives and analytical alternatives were equally frequent in their sources, while the analytical prepositional constructions dominated from the fourteenth century onwards, paralleling the shift from synthetic genitives to *of*-constructions in Middle English (cf. Weerman and de Wit 1999: 1159 citing Mustanoja 1960).

While case and gender remained core issues in Dutch grammar books from the seventeenth and eighteenth centuries, some significant changes can be discerned. Metalinguistic discourse at the beginning of the eighteenth century was fairly elitist, targeting a socially privileged audience of primarily ministers and literary authors, i.e. a largely male audience well-versed in classical grammar (Rutten 2009). From c. 1740 onwards, the target audience was increasingly conceptualised more broadly, until it included all the members of the Dutch nation in the final decades of the eighteenth century. Similar changes were going on across Europe, see for example Yáñez-Bouza (2018: 31–32) for eighteenth-century English. This change from an elitist orientation to eventually grammar as a matter of national concern signaled the increasing importance of the ‘national’ culture and is one indication of the spread of standard language ideology.

The new, ‘national’ approach to grammar, which aimed to reach the entire population, was characterised by a variety of didactic impulses such as easier, that is Dutchified terminology, and syntactically less complex language (Rutten 2009). Another important difference compared with the earlier elitist approach to grammar consisted in a changing attitude towards nominal case (Rutten 2012). The most important evolution relevant here concerns the prescriptions found in the grammatical tradition for the genitive, dative and ablative cases. For example, grammarians mostly preferred the historically present synthetic genitive case at the beginning of the eighteenth century, in the so-called elitist period. The genitive masculine singular of the definite article, for example, was *des*, consisting of *de* ‘the’ and the case ending *-s*. In the course of the century, more and more grammarians mention the analytical alternative *van den*, consisting of the preposition *van* ‘of’ and *de* ‘the’ plus the case ending *-n*. Note that the nominal cases did not all disappear at once: the genitive was the first to decline in the late Middle Ages, while the dative and accusative, though often inseparable due to syncretism, survived longer. The *n*-inflection on the article, therefore, should not be seen as one case ending (*-n*) replacing another (*-s*). Instead, the genitive case was on the way out, and genitival functions were taken over by prepositional phrases, often with *van*, which could still trigger dative and accusative endings. In addition, *den* and the uninflected form *de* were also subject to syncretism due to vowel reduction and *n*-deletion, two phonetic changes characteristic of Middle and Early Modern Dutch.

Thus, prepositional genitival constructions were on the rise, not only in language use, but by the second half of the eighteenth century also in prescription, which is an indication that metalinguistic discourse was increasingly targeting the entire language community. However, when the discourse about the importance of a ‘national’ grammar led to an actual publication with that status (Weiland 1805), the former, elitist preference for synthetic genitives was revived, and analytical alternatives with *van* were demoted again, and in any case not deemed suitable for more formal or ‘elevated’ types of written Dutch (see Rutten 2012 for discussion). In the wake of Weiland (1805), a range of new grammar books came on the market, many of which were specifically meant for use in education. Most of these followed Weiland’s preference for the synthetic genitive, thus prescribing forms such as *des* and *der* in the masculine/neuter and feminine singular, while mentioning prepositional phrases such as *van de* merely as alternatives, if they were mentioned at all (Rutten 2016a).

The use of the synthetic genitive also depends on nominal gender and number, as Scott (2014: 121f.) points out striking differences between masculine and neuter singular forms (e.g. *des*) on the one hand, and feminine singular and plural forms of all genders (e.g. *der*) on the other. The latter group appeared to have outnumbered the former by the nineteenth century.

In summary, the discrepancy between the norms promoted in metalinguistic discourse from the sixteenth century onwards on the one hand, and actual language use from the Middle Ages onwards on the other is extreme in the case of genitival constructions in Dutch (cf. Vezzosi 2000). As such, the topic merits detailed corpus-based research in order to assess the relationship between prescription and language practice. The period around 1800 is particularly interesting, since the ‘national’ grammar of 1805 broke with the eighteenth-century prescriptive evolution towards analyticity and instead conservatively promoted the synthetic forms, thereby enlarging again the breach between prescription and practice.

## 4 Corpus and methodology

In order to examine the effects of historical prescriptivism and, more concretely, prescriptive norms in the context of the Dutch national language policy around 1800 on actual language practices, we compiled a multi-genre corpus of late eighteenth- and early nineteenth-century Dutch. The Going Dutch Corpus comprises 421,878 words in total. Taking the *schrijftaalregeling* of 1804/1805 as the point of departure, the two diachronic cross-sections of the corpus, viz. 1770–1790 (period 1) and 1820–1840 (period 2), represent the generations of language users before and after the official language regulations were introduced.

Allowing for the fact that diachronic changes may affect different genres to different extents, the Going Dutch Corpus incorporates three genres: (1) private letters, (2) diaries and travelogues, and (3) newspapers. Following the research tradition on language histories ‘from below’, the corpus includes two types of handwritten ego-documents (cf. Elspaß et al. 2007, van der Wal and Rutten 2013). First, private letters were selected as the written sources closest to oral language for historical-sociolinguistic research (Nevalainen and Raumolin-Brunberg 2016). Secondly, we added unpublished diaries and travelogues as another type of ego-document, which are often closer to supralocal writing traditions than private letters (Schneider 2013). As a third genre, we included newspapers, which represent published and edited language, while they were still locally produced and distributed. The inclusion of newspapers also allows a comparison between manuscript and print. All texts in the Going Dutch Corpus were manually transcribed from digital images of original archive sources (cf. Krogull 2018b: ch. 4 for a discussion of our corpus methodology).

**Figure 1:**
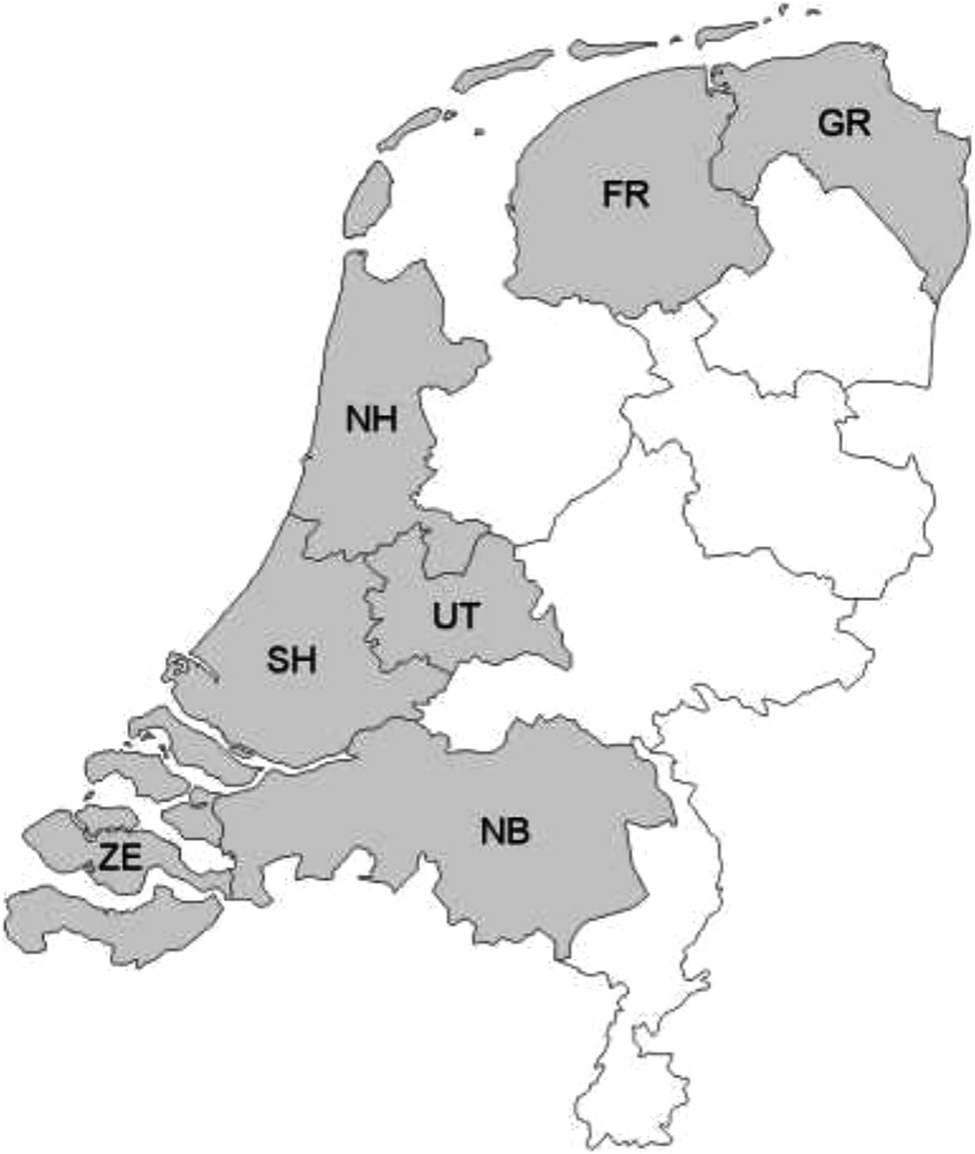
Map of the Netherlands indicating the regions represented in the Going Dutch Corpus (FR = Friesland, GR = Groningen, NB = North Brabant, NH = North Holland, SH = South Holland, UT = Utrecht, ZE = Zeeland).

Geographically, for all genres, the Going Dutch Corpus covers seven regions of the northern Low Countries: Friesland, Groningen, North Brabant, North Holland, South Holland, Utrecht, and Zeeland (see Figure 1 for a map). These regions were chosen on several grounds. North and South Holland are traditionally considered the demographic and economic centre of the Dutch language area, at least from the seventeenth century onwards. Previous large-scale historical-sociolinguistic analyses of Dutch have focused strongly on the Holland and Zeeland areas (Rutten and van der Wal 2014). The present research both continues and broadens this previous research by also including a layer of northern and central regions, viz. Groningen, Friesland, Utrecht and North Brabant.

The ego-documents also incorporate gender as a social variable. Although texts by male writers constitute two-thirds of the corpus, we consider the proportion of texts by female writers (i.e. one-third3For private letters, a well-balanced gender representation (54% men, 46% women) was achieved. Due to the relative scarcity of diaries and travelogues by women, however, the majority of these texts in our corpus was written by men (84%).) as a substantial change with respect to the near-absence of women in traditional language histories. In terms of socio-economical groups, most ego-documents in the corpus were produced by members of the middle to the upper ranks of society, excluding the very highest rank (see Rutten and van der Wal 2014: 9–10 for the seventeenth- and eighteenth-century social stratification).

In sum, the Going Dutch Corpus represents two periods, three genres, seven regions, and, in the case of ego-documents, two genders. The ego-documents comprise 400 private letters, written by 298 individual informants, and 50 diaries and travelogues by 50 writers. Table 2 presents the make-up of the corpus. All linguistic examples presented in this section are taken from the Going Dutch Corpus.

**Table 2:** The Going Dutch Corpus.

		Period 1 1770–1790	Period 2 1820–1840	Total
Genre	Private letters	105,427	105,299	210,726
	Diaries and travelogues	71,157	69,350	140,507
	Newspapers	35,323	35,322	70,645
		**211,907**	**209,971**	**421,878**
Region	Friesland	30,757	30,949	61,706
	Groningen	28,875	30,323	59,198
	North Brabant	30,647	25,998	56,645
	North Holland	30,256	32,368	62,624
	South Holland	30,225	33,547	63,772
	Utrecht	30,588	30,094	60,682
	Zeeland	30,559	26,692	57,251
		**211,907**	**209,971**	**421,878**
Gender	Male	127,112	105,657	232,769
	Female	49,472	68,992	118,464
		**176,584**	**174,649**	**351,233**

For the analysis of genitival constructions in late eighteenth- and early nineteenth-century Dutch, we focus on the synthetic (adnominal) genitive case, both in prenominal and post-nominal position (see (1) and (2), respectively), and its strongest competitor, i.e. the analytical construction with the preposition *van* ‘of’ (see (3)).

(1)

**Table:** 

*des*	*Heeren*	*aanhoudenden*	*zegen*
The+GEN	Lord+GEN	constant	blessing
‘the constant blessing of the Lord’

(2)

**Table:** 

*de*	*andere zyde der*	*stad*
the	other side the+GEN	city
‘the other side of the city’

(3)

**Table:** 

*in*	*het*		*midden*	*van de*	*kerk*
in	the	middle		of the	church
‘in the middle of the church’					

Two more prenominal alternatives, viz. the possessive s-construction (see (4)) and the periphrastic possessive *z’n*-construction (see (5)), will not be discussed in this paper (cf. Nobels 2013; Simons 2013; Scott 2014; Weerman and de Wit 1999).

(4)

**Table:** 

*onse*	*dierbaare*	*Moeders*	*ziekte*
our	dear	Mother+GEN	illness
‘our dear mother’s illness’

(5)

**Table:** 

*de*	*kaptyn*	*syn*	*dochter*
the	captain	his	daughter
‘the captain’s daughter’			

Unlike the analytical option with *van*, both the *s-*construction and the *z’n*-construction first and foremost occur with animate and, more specifically, human possessors (Scott 2014: 103), although counterexamples can certainly be found. However, in many instances with inanimate possessors, these two alternative constructions cannot replace the synthetic genitive case. The *van*-construction does not have those semantical and functional restrictions, which is why we consider it the only fully interchangeable alternative of the historical genitive case in the sense of a sociolinguistic variable.

Making use of the entire Going Dutch Corpus, we extracted the occurrences of various genitive markers and their analytical counterparts with the preposition *van*, focusing on definite and indefinite articles *(de* ‘the’, *het* ‘the’, *een* ‘a’*),* demonstrative pronouns *(deze* ‘this, these’, *dit* ‘this’, *die* ‘that, those’*)* and possessive pronouns *(mijn* ‘my’, *ons* ‘our’, *zijn* ‘his’, *haar* ‘her’, *hun* ‘their’, *uw* ‘your’). Masculine and neuter singular genitive markers are identical, as are feminine singular and all plural forms. See Table 3 for an overview (cf. also Scott 2014: 122).

**Table 3:** Genitive markers and their analytical counterpart with *van*.

	Genitive case	*van*-construction	Translation
Articles (definite, indefinite)	*des (‘s)* (m./n.),	*van den* (m.)*, van het (‘t)* (n.),	of the, these
	*der* (f./plur.)	*van de* (f./plur.)	
	*eens* (m./n.),	*van eenen* (m.)*, van een* (n.)*,*	of a
	*eener* (f./plur.)	*van eene* (f./plur.)	
Demonstrative pronouns	*dezes* (m./n.)*,*	*van dezen* (m.)*, van dit* (n.)*,*	of this, these
	*dezer* (f./plur.)	*van deze* (f./plur.)	
	*diens* (m./n.)*,*	*van dien* (m.)*, van dat* (n.)*,*	of that, these
	*dier* (f./plur.)	*van die* (f./plur.)	
Possessive pronouns	*mijns* (m./n.),	*van mijnen* (m.)*, van mijn* (n.)*,*	of my
	*mijner* (f./plur.)	*van mijne* (f./plur.)	
	*onzes* (m./n.),	*van onzen* (m.)*, van ons* (n.)*,*	of our
	*onzer* (f./plur.)	*van onze* (f./plur.)	
	*zijns* (m./n.),	*van zijnen* (m.)*, van zijn* (n.)*,*	of his
	*zijner* (f./plur.)	*van zijne* (f./plur.)	
	*haars* (m./n.),	*van haren* (m.)*, van haar* (n.)*,*	of her
	*harer* (f./plur.)	*van hare* (f./plur.)	
	*huns* (m./n.),	*van hunnen* (m.)*, van hun* (n.)*,*	of their
	*hunner* (f./plur.)	*van hunne* (f./plur.)	
	*uws* (m./n.),	*van uwen* (m.)*, van uwe* (n.)*,*	of your
	*uwer* (f./plur.)	*van uwe* (f./plur.)	

A number of undesired occurrences were categorically filtered out by hand, such as the absolute genitive *(*e.g. *des winters* ‘in the winter’), the partitive genitive *(*e.g. *de meeste hunner* ‘most of them’), and fixed expressions with a genitive (e.g. *des noods* ‘if need be’). We also excluded proper names with *van de(n)* (e.g. *de heer van de Capelle* ‘Mr van de Capelle’), phrasal verbs with *van* (e.g. *afscheid nemen van* ‘say farewell to’) and temporal markers of the type *van de week* ‘in the course of this week’. Furthermore, we considered possible spelling variation such as <e>/<ee>, <ij>/<y>, <s>/<z> and so forth.

The occurrences selected for this case study cover a variety of specific and more fixed contexts. Previous historical-sociolinguistic research on the Dutch genitive, mainly based on seventeenth- and eighteenth-century private letters, has attested that “context is a major factor of influence in the distribution of the genitive case and alternative constructions” (Nobels and Rutten 2014: 40). Especially for letter writing, it is generally claimed that historical forms such as the genitive case are more likely to be preserved in formulaic contexts than in more neutral, non-formulaic contexts (Simons and Rutten 2014: 65). Particularly the introduction and the ending of a prototypical letter structure tend to be composed of recurring formulae (Rutten and van der Wal 2014: ch. 3).

In this paper, we therefore distinguish neutral from various specific contexts, viz. dates (see example (6)), religious formulae (see (7))*,* as well as other (i.e. non-religious) formulae, such as epistolary formulae (see (8)) or fixed expressions (see (9))*.* Additionally, we consider prepositional expressions like *uit hoofde* (+gen./*van*) ‘by reason of’ as a separate context (see (10)).

(6)

**Table:** 

*den*	*28sten*	*der*	*vorige*	*maand*
the	28^th^	the+GEN	previous	month
‘the 28^th^ of the previous month’

(7)

**Table:** 

*de*	*byzondere*	*gunst*	*en*	*goedheid*	*des*	
the	extraordinary	mercy	and	goodness	the+GEN	
*Allerhoogsten*						
Almighty+GEN						
‘the extraordinary mercy and goodness of the Almighty’

(8)

**Table:** 

*onder*	*het*	*schrijven*	*deses*
under	the	writing	this+GEN
‘while writing this [letter]’

(9)

**Table:** 

*eene*	*dezer*	*daagen*
one	these+GEN	days
‘one of these days’

(10)

**Table:** 

*uit*	*hoofde*	*d* *er*	*grote*	*warmte*
by	head	the+GEN	great	heat
‘by reason of/in consideration of the (great) heat’				

In total, our corpus search generated 4,762 tokens of genitival constructions, distributed across the synthetic genitive case and its alternative *van*-construction. The findings are presented and discussed in section 5.

## 5 Results

### 5.1 Diachrony and context

Seeking to assess the potential influence of Weiland’s (1805) officialised prescription in favour of the synthetic genitive, Table 4 provides a diachronic overview of the two genitival constructions under investigation.

**Table 4:** Distribution between Period 1 and Period 2.

	Period 1 (1770–1790)		Period 2 (1820–1840)	
	N	%	N	%
Synthetic	871	36.0	969	41.4
Analytical	1,548	64.0	1,374	58.6
Total	2,419	100	2,343	100

The distribution of synthetic and analytical options turns out to be relatively stable across time. In the late eighteenth-century data, the prepositional *van*-construction (64.0%) clearly outweighs the historical genitive forms (36.0%), which is also the case in the early nineteenth-century data, i.e. after the prescription of 1805. However, Table 4 also displays a slight increase of the synthetic option in period 2 from 36.0 to 41.4%, which might signal an effect of the national grammar by Weiland. Recall that from 1805 onwards, language users can be expected to have gone through the national education system, where they were exposed to the official standard norms.4While this is not necessarily the case for the ‘pre-Weiland’ generations of adult writers, Krogull (2018b: ch. 13) presents evidence that older language users also adopted prescribed forms (at least on the level of orthography). This signals that (school) education was not the only means of transmitting written standard norms. However, the developments in language practice call for a more fine-grained analysis, which is why we examine a range of internal and external factors in this section.

First, we address the role of context. In line with earlier observations, the results in Table 5, based on our Going Dutch Corpus data, indicate a considerable amount of context-related variation.

**Table 5:** Distribution across context between Period 1 and Period 2.

	Period 1 (1770–1790)		Period 2 (1820–1840)	
	Synthetic % (N)	Analytical % (N)	Synthetic % (N)	Analytical % (N)
Neutral	30.5 (569)	69.5 (1,297)	41.3 (819)	58.7 (1,165)
Dates	62.1 (105)	37.9 (64)	43.8 (63)	56.3 (81)
Religious formulae	76.9 (113)	23.1 (34)	64.7 (11)	35.3 (6)
Other formulae	41.0 (50)	59.0 (72)	51.7 (30)	48.3 (28)
Prepositional expressions	29.6 (34)	70.4 (81)	32.9 (46)	67.1 (94)

To begin with, neutral contexts, which constitute by far the most common type of context, clearly prefer the analytical construction with *van* in period 1 (69.5%). Interestingly, the less frequent synthetic option gains ground in period 2 (from 30.5 to 41.3%), which confirms the tendency already revealed in Table 4 and potentially signals normative influence in the direction of Weiland’s prescription.

With regard to the more formulaic types of context, religious formulae are still fairly common in the eighteenth-century period and mostly occur with the genitive (76.9%). Diachronically, however, we can observe a strong decrease in absolute numbers with no more than 17 instances in the nineteenth-century data, from which we conclude that religious formulae no longer played a major role. The other formulae are more or less balanced in terms of synthetic and analytical constructions, especially in period 2. The distribution in prepositional expressions, largely preferring the *van*-construction (around 70%), is stable across time.

The most remarkable results are found in the context of dates, showing a decrease of synthetic forms. While the historical genitive is the most frequently used option in period 1 (62.1%), the *van*-construction becomes prevalent in period 2 (56.3%). In order to find a possible explanation for this pattern, we zoomed in on the use of dates across genre. It shows that the distribution of synthetic and analytical options is fairly well-balanced in private letters, where it remains stable across the two periods. In diaries and travelogues, the number of tokens is surprisingly low, already in the eighteenth century, and thus hardly affects the overall distribution. The changing pattern, in fact, can only be observed in newspapers. In these texts, the dominant synthetic option in period 1 (73.2%) appears to be replaced by the analytical alternative in period 2 (57.0%). On closer inspection, the context of dates appears to comprise two different types, viz. (1) the often elliptical construction of the type *den 22 dezer [Maand]* ‘the 22nd of this [month]’, and (2) references to the date of particular events like *de aardbeving van den 26 Nov.* ‘the earthquake of 26^th^ November’. Diachronically, the former decreases in absolute terms, while the latter gains ground and clearly prefers the analytical option. There are thus no indications that the analytical option actually replaced the synthetic genitive in the first (more formulaic) type of dates.

### 5.2 Genre, region and gender

Having confirmed the relevant role of context in the choice of genitival constructions, we henceforth concentrate on occurrences in the neutral context, which represent the most creative (=non-formulaic) language use. We discuss three external factors integrated in our corpus design, viz. genre, region and gender, before we address the internal factor of forms (see section 5.3).

#### 5.2.1 Genre variation

**Figure 2:**
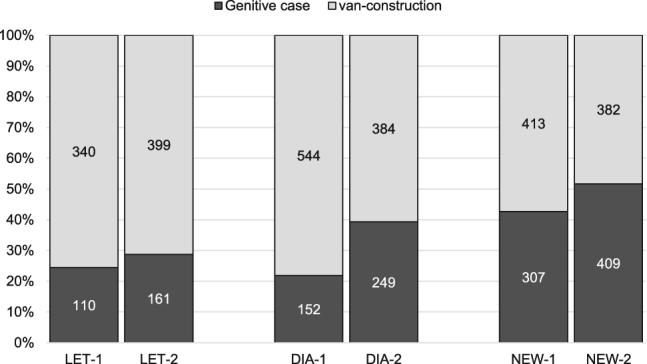
Distribution across genre between Period 1 and Period 2.

Focusing on the distribution across genre, Figure 2 reveals that the synthetic genitive case gains ground across all three genres of the Going Dutch Corpus, both handwritten (LET = private letters, DIA = diaries and travelogues) and printed (NEW = newspapers).

This is a strikingly coherent development against the background of Weiland’s conservative endeavour to ‘revive’ the historical case system. In private letters, i.e. the most ‘oral’ and informal genre in our corpus, the synthetic construction increases modestly from 24.4% in period 1 to 28.8% in period 2. Evidently, the analytical alternative with *van* remains the prevalent choice in these sources. The rise of the genitive is more pronounced in the other two genres, though. In diaries and travelogues, the synthetic option increases from a relatively low 21.8% in period 1 to 39.3% in period 2. In newspapers, the genitive case is already fairly frequent in period 1 (42.6%), but gains even more ground in period 2 (51.7%), where is occurs alongside the more or less equally frequent *van*-construction (48.3%).

#### 5.2.2 Regional variation

**Figure 3:**
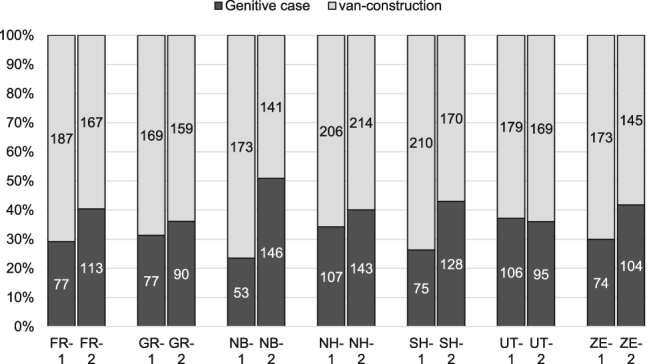
Distribution across region between Period 1 and Period 2.

Investigating possible geographical variation in the use of genitival constructions, Figure 3 displays the distribution across region (FR = Friesland, GR = Groningen, NB = North Brabant, NH = North Holland, SH = South Holland, UT = Utrecht, ZE = Zeeland), in neutral contexts.

In the late eighteenth-century period, the analytical option outnumbers the genitive case in all seven regions. Synthetic forms are particularly rare in North Brabant (23.5%), whereas they are most common in Utrecht (37.2%). Diachronically, however, the results across region attest the increase of synthetic genitive forms across all regions, except for the region of Utrecht (where the distribution is more or less stable). Most remarkable is the rise of the genitive in North Brabant from 23.5 to 50.9%, thus as frequent as the analytical option in period 2. Interestingly, this development in the North Brabant data is not restricted to one specific type of sources, but can be observed for all three genres of the corpus.

#### 5.2.3 Gender variation

**Figure 4:**
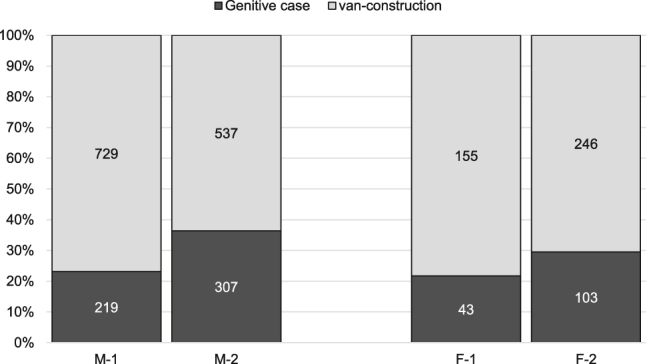
Distribution across gender between Period 1 and Period 2.

Next, we also examined the social variable of gender (M = male, F = female) in neutral context, as presented in Figure 4.

In the eighteenth-century data, there are practically no gender differences in the use of genitival constructions, with both genders preferring the analytical option in 76.9% (men) and 78.3% (women). The nineteenth-century results indicate a diachronic increase of synthetic forms, gaining ground in ego-documents written by men (from 23.1 to 36.4%) and, to a slightly lesser extent, women (from 21.7 to 29.5%).

In sum, the corpus-based results in this section thus testify to an increase of the genitive case in early nineteenth-century language practice across all genres, almost all regions and both genders, at least in neutral contexts.

### 5.3 Internal factor: forms of markers

Having investigated the influence of three external factors, we address one internal factor that has been claimed to affect the use of genitival constructions considerably. Depending on nominal gender and number, genitive markers can take different forms, for instance *des*, *eens*, *mijns* for masculine and neuter singular nouns, as opposed to *der*, *eener*, *mijner* for feminine singular and plural nouns of all genders (see Table 3 for an overview). With regard to the token frequency of nouns in the genitive, Scott (2014: 121f.), amongst others, observes striking differences between the masculine and neuter singular forms like *des* on the one hand, and feminine singular and plural forms of all genders like *der* on the other. By the nineteenth century, the latter group had outnumbered the former considerably.

**Figure 5:**
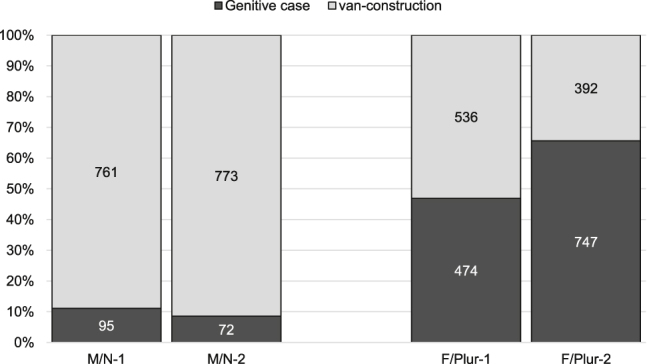
Distribution across form between Period 1 and Period 2.

The question arises whether and to what extent the effectiveness of Weiland’s official prescription was thus also dependent on the specific form and frequency of genitive markers. Figure 5 displays the distribution of synthetic and analytical options across masculine and neuter (M/N, i.e. forms such as *des*), as well as feminine and plural forms (F/Plur, i.e. forms such as highly frequent *der*), in neutral context.

These findings reveal marked form-related differences. For masculine and neuter singular forms, the analytical *van-*construction is already established as the most frequent option by the late eighteenth century (88.9%) and further consolidates its preponderance in the early nineteenth century (91.5%). In other words, synthetic genitive forms are already marginal in period 1 and continue to disappear from language practice in period 2, in spite of Weiland’s prescriptive norm advocating the use of case inflections.

For feminine singular and all gender plural forms, our results give a completely different picture, though. In period 1, both genitival constructions are evenly distributed (approximately 50/50). Remarkably, we can witness a steep increase of the synthetic genitive in feminine and plural forms in period 2 (from 46.9 to 65.6%), at the expense of the analytical alternative. At least for feminine and plural forms, the developments in language use suggest normative influence.

Pointing out that “most nouns occurring in the genitive were feminine singulars and plurals of all genders”, Scott (2014: 121) considers the higher token frequency of these genitive markers as a crucial internal factor. Generally, our findings based on the Going Dutch Corpus confirm this major difference in token frequency of genitive markers (i.e. 95 occurrences M/N versus 474 occurrences F/Plur in period 1; 72 M/N versus 747 F/Plur in period 2). Scott (ibid.) further argues that “[t]he high token frequency of feminine singular and all genders plural nouns in the genitive in the 19^th^ century may well have aided the preservation of the *x der y* structure but not a masculine/neuter equivalent”. Indeed, *x der y* (with the particularly frequent definite article *der*) was the structure with the highest absolute token frequency and had therefore reached the highest familiarity among language users (Scott 2014: 121–122). Nevertheless, our corpus results give evidence that the rise of feminine and plural genitive markers in the early nineteenth century is not restricted to the *x der y* structure. In fact, the increase of synthetic forms in period 2 can be attested not only for the definite article *der* (from 47.0 to 67.2%), but also for feminine and plural forms of the indefinite article *eener* (from 44.4 to 59.1%) and possessive pronouns (from 47.7 to 60.4%). While this does not categorically rule out the special role of the *x der y* structure and its ‘preserving effect’, the increase of synthetic forms affected more genitive makers than *der* alone, probably indicating normative influence. No such effect could be witnessed for masculine and neuter genitive markers, all of which were largely replaced by the analytical construction with *van*.

## 6 Discussion and conclusions

In this paper, we analysed the success of the first Dutch national language policy on patterns of language use. The linguistic variable that we focused on was the genitive case. On the one hand, the genitive case is a relatively complex grammatical feature, especially given the fact that it is commonly assumed to have vanished from the spoken language centuries earlier, rendering it an exogeneous form that had to be acquired through explicit instruction. On the other hand, centuries of metalinguistic discourse had promoted the genitive case, and it remained in use in the written language so that language users were likely to be confronted with the genitive case when reading. The discrepancy between a grammatical category close to death on the one hand, and continued prescriptive efforts to revive it on the other makes the Dutch genitive case an exciting case for a study of the effects of top-down language planning.

Assessing such effects on language use, our corpus results suggest that the conservative prescription in Weiland’s national grammar of Dutch could indeed boost the use of feminine singular and all gender plural genitive markers. It is likely that a combination of the prescribed genitive forms in Weiland on the one hand, and a generally high familiarity among language users on the other hand (see below), both contributed to the increase of feminine singular and all gender plural genitive markers in the early nineteenth century. The increase of synthetic genitives in almost all regions and in the writings of both men and women also testifies to the overall success of the prescription and this generally high familiarity. Equally important is the fact that the frequency of the genitive case also gained ground in neutral contexts in private letters. This is arguably the most informal context in the corpus used, and even there synthetic genitives gain ground. At the same time, we showed that the prescription failed to revive the genitive case in masculine and neuter singular markers. These forms had already been infrequent in absolute terms by the end of the eighteenth century and were no longer familiar to most language users. We argue that this is why the genitive in these forms could not be revived effectively.

As in our earlier study of the effect of Weiland’s 1805 prescriptions on the distribution of relativisers (Krogull et al. 2017), we established important genre differences. The increase of the genitive in neutral context in private letters is remarkable, but much more significant is the increase in diaries and newspapers. There was a clear genre-specific difference in terms of sensibility to linguistic prescription, and it is important to stress that diaries were quite different (i.e. less ‘oral’, closer to supralocal writing traditions) from the other type of ego-document used, viz. private letters.

The results generally signal a normative ‘Weiland effect’ on the use of the Dutch genitive case, at least to a certain degree and with some internal restrictions. Importantly, though, the observed effectiveness of official prescriptions with respect to the genitive case is fairly subtle compared to the more drastic developments attested for various orthographic variables, coherently shifting in the direction of the official spelling rules by Siegenbeek (1804) in a short period of time (Krogull 2018a, 2018b; Rutten et al. 2020). Examples include the shift from <d> to <dt> for final /t/ in *d*-stem verbs (e.g. *hij vindt* ‘he finds’), the etymologically motivated split of syllable-final /xt/ into <cht> and <gt> (e.g. *dacht* ‘thought’ versus *bragt* ‘brought’), and the phonology-based system of <ee> and <e> for distinct long *e*’s in open syllable (e.g. *deelen* ‘share’ versus *geven* ‘give’). From this we can conclude that the effectiveness of linguistic prescriptions on language practice needs to be assessed on at least two distinct levels, viz. orthography and grammar (cf. Rutkowska and Rössler 2012: 213).

While this might not be an entirely surprising conclusion as such, our findings are based on reliable empirical data drawn from a large-scale corpus of historical Dutch, showing that grammatical issues are much less sensible to top-down policy measures than orthography, and often further conditioned by both internal and external factors.

However, when we compare the genitive with the similarly intriguing morphosyntactic case of relative pronouns (Krogull et al. 2017), differences in the Dutch grammatical tradition become apparent. On the one hand, a considerable amount of metalinguistic attention has been devoted to the genitive case for many centuries, which suggests a high awareness of this issue. Therefore, it seems not unlikely that language users encountered metalinguistic commentary surrounding the genitive, and thus develop some awareness of its social significance. On the other hand, a normative tradition of such historical depth does not exist for relative pronouns at all. In fact, prior to Weiland’s 1805 grammar, relativisation was not among the core topics in metalinguistic discourse and was in fact only sporadically commented on in normative works. In the case of the genitive, Weiland’s (1805) intervention continued an established tradition of metalinguistic reflection, whereas his interest in relativisers was relatively new. This difference in metalinguistic awareness may have contributed to the differing success of Weiland’s prescriptions for the genitive and for relativisers, respectively, albeit that the success was still small in the case of the genitive, too.

We have shown that the prescription in favour of synthetic forms does not affect the use of the genitive *tout court*, but is limited to feminine and all gender plural markers like *der*. This can be explained by the frequency of the various forms before the time of the prescription. Synthetic options in masculine and neuter forms such as *des* were already marginal in the late eighteenth century and decreased in the first half of the nineteenth century, thereby going entirely against the grain of the prescribed norm of 1805. Feminine and plural forms such as *der* made up around 50% of the tokens in the late eighteenth century and seem to have been still prominent enough in language use to be susceptible to prescriptive influence. An important question for future research will therefore be whether there is a frequency threshold for prescriptive success, i.e. a minimal frequency of prescribed forms in actual language in order to successfully promote these forms.

In this paper, we have studied the effectiveness of top-down language planning on patterns of language use from a corpus-based perspective in an effort to operationalise implementation and acceptance in the sense of Haugen (1966, 1987). Our generalisations concern differences between genres and between spelling and grammar. Within the domain of grammar, the level of social awareness and the frequency of the features involved seemed to influence the effectiveness of prescriptions. These generalisations would ideally be tested through empirical analyses of the success of language planning in other language areas. This would contribute to our knowledge of standardisation as a multilayered historical phenomenon.

## References

[j_jhsl-2019-0016_ref_001] Anderwald Lieselotte (2016). *Language between description and prescription. Verbs and verb categories in nineteenth-century grammars of English*.

[j_jhsl-2019-0016_ref_002] Audring Jenny (2006). Pronominal gender in spoken Dutch. *Journal of German Linguistics*.

[j_jhsl-2019-0016_ref_003] Dibbets Geert (1995). *De woordsoorten in de Nederlandse triviumgrammatica* [Parts of speech in the Dutch trivium grammar].

[j_jhsl-2019-0016_ref_004] Elspaß Stephan, Langer Nils, Scharloth Joachim, Vandenbussche Wim (2007). *Germanic language histories ‘from below’ (1700–2000)*.

[j_jhsl-2019-0016_ref_005] Geerts Guido (1966). *Genus en geslacht in de Gouden Eeuw* [Natural and grammatical gender in the Golden Age].

[j_jhsl-2019-0016_ref_006] Haugen Einar (1966). Dialect, language, nation. *American Anthropologist*.

[j_jhsl-2019-0016_ref_007] Haugen Einar (1987). *Blessings of Babel. Bilingualism and language planning. Problems and pleasures*.

[j_jhsl-2019-0016_ref_008] Krogull Andreas (2018a). De effectiviteit van de schrijftaalregeling. Historisch-sociolinguïstisch onderzoek naar taalbeleid en taalgebruik in de achttiende en negentiende eeuw [The effectiveness of the ‘written language regulation’. Historical-sociolinguistic research on language policy and language practice in the eighteenth and nineteenth century]. *Internationale Neerlandistiek*.

[j_jhsl-2019-0016_ref_009] Krogull Andreas (2018b). *Policy versus practice. Language variation and change in eighteenth- and nineteenth-century Dutch*.

[j_jhsl-2019-0016_ref_010] Krogull Andreas, Rutten Gijsbert, van der Wal Marijke, Säily Tanja, Nurmi Arja, Palander-Collin Minna, Auer Anita (2017). Relativisation in Dutch diaries, private letters and newspapers (1770-1840): A genre-specific national language?. *Exploring future paths for historical sociolinguistics*.

[j_jhsl-2019-0016_ref_011] Leerssen Joep (2018a). *Encyclopedia of Romantic Nationalism in Europe*.

[j_jhsl-2019-0016_ref_012] Leerssen Joep, Leerssen Joep (2018b). Introduction. *Encyclopedia of romantic nationalism in Europe*.

[j_jhsl-2019-0016_ref_013] Lippi-Green Rosina (2012). *English with an accent: Language, ideology, and discrimination in the United States*.

[j_jhsl-2019-0016_ref_014] Loey Adolphe van (1980). *Middelnederlandse spraakkunst. I. Vormleer* [Middle Dutch grammar. I. Morphology].

[j_jhsl-2019-0016_ref_015] McLelland Nicola., Rutten Gijsbert, Vosters Rik, Vandenbussche Wim (2014). Language description, prescription and usage in seventeenth-century German. *Norms and usage in language history, 1600–1900. A sociolinguistic and comparative perspective*.

[j_jhsl-2019-0016_ref_016] Milroy James, Milroy Lesley (2012). *Authority in language. Investigating standard english*.

[j_jhsl-2019-0016_ref_017] Mustanoja Tauno (1960). *A Middle English syntax*.

[j_jhsl-2019-0016_ref_018] Nevalainen Terttu, Raumolin-Brunberg Helena (2016). *Historical sociolinguistics. Language change in Tudor and Stuart English*.

[j_jhsl-2019-0016_ref_019] Nobels Judith (2013). *(Extra)Ordinary letters. A view from below on seventeenth-century Dutch*.

[j_jhsl-2019-0016_ref_020] Nobels Judith, Rutten Gijsbert, Rutten Gijsbert, Vosters Rik, Vandenbussche Wim (2014). Language norms and language use in seventeenth-century Dutch. Negation and the genitive. *Norms and usage in language history. A sociolinguistic and comparative perspective*.

[j_jhsl-2019-0016_ref_021] Poplack Shana, Lidia-Gabriela Jarmasz, Dion Nathalie, Rosen Nicole (2015). Searching for standard French: The construction and mining of the *Recueil historique des grammaires du français*. *Journal of Historical Sociolinguistics*.

[j_jhsl-2019-0016_ref_022] Quak Arend, van der Horst Joop M. (2002). *Inleiding Oudnederlands [Introduction to Old Dutch]*.

[j_jhsl-2019-0016_ref_023] Rutkowska Hanna, Paul Rössler, Hernández-Campoy Juan Manuel, Conde-Silvestre Juan Camilo (2012). Orthographic variables. *The handbook of historical sociolinguistics*.

[j_jhsl-2019-0016_ref_024] Rutten Gijsbert (2009). Grammar to the people. The Dutch language and the public sphere in the 18th century: With special reference to Kornelis van der Palm. *Beiträge zur Geschichte der Sprachwissenschaft*.

[j_jhsl-2019-0016_ref_025] Rutten Gijsbert (2012). ‘Lowthian’ linguistics across the North Sea. *Historiographia Linguistica*.

[j_jhsl-2019-0016_ref_026] Rutten Gijsbert (2016a). Teaching the genitive. Variation of genitival constructions in Dutch ‘national’ grammar (1800–1830). *Beiträge zur Geschichte der Sprachwissenschaft*.

[j_jhsl-2019-0016_ref_027] Rutten Gijsbert (2016b). Standardization and the myth of neutrality in language history. *International Journal of the Sociology of Language*.

[j_jhsl-2019-0016_ref_028] Rutten Gijsbert (2019). *Language planning as nation building. Ideology, policy and implementation in the Netherlands*.

[j_jhsl-2019-0016_ref_029] Rutten Gijsbert, Krogull Andreas, Schoemaker Bob (2020). Implementation and acceptance of national language policy. The case of Dutch (1750–1850). *Language Policy*.

[j_jhsl-2019-0016_ref_030] Rutten Gijsbert, Vosters Rik, Vandenbussche Wim (2014). *Norms and usage in language history. A sociolinguistic and comparative perspective*.

[j_jhsl-2019-0016_ref_031] Rutten Gijsbert, van der Wal Marijke (2014). *Letters as loot. A sociolinguistic approach to seventeenth- and eighteenth-century Dutch*.

[j_jhsl-2019-0016_ref_032] Schneider Edgar W, Chambers Jack K., Schilling Natalie (2013). Investigating historical variation and change in written documents. New perspectives. *The Handbook of language variation and change*.

[j_jhsl-2019-0016_ref_033] Schoemaker Bob (2018). *Gewijd der jeugd, voor taal en deugd. Het onderwijs in de Nederlandse taal op de lagere school, 1750-1850* [Dutch language education in primary schools, 1750–1850.

[j_jhsl-2019-0016_ref_034] Schoemaker Bob, Rutten Gijsbert (2017). Standard language ideology and Dutch school inspection reports (1801–1854). *Sociolinguistica*.

[j_jhsl-2019-0016_ref_035] Scott Alan K. (2014). *The genitive case in Dutch and German: A study of morphosyntatic change in codified languages*.

[j_jhsl-2019-0016_ref_036] Siegenbeek Matthijs (1804). *Verhandeling over de Nederduitsche spelling, ter bevordering van eenparigheid in dezelve* [Treatise of the Dutch spelling].

[j_jhsl-2019-0016_ref_037] Simons Tanja (2013). *Ongekend 18e-eeuws Nederlands. Taalvariatie in persoonlijke brieven* [Unknown 18th-century Dutch. Language variation in personal letters].

[j_jhsl-2019-0016_ref_038] Simons Tanja, Rutten Gijsbert, Rutten Gijsbert, Vosters Rik, Vandenbussche Wim (2014). Language norms and language use in eighteenth-century Dutch: Final n and the genitive. *Norms and usage in language history. A sociolinguistic and comparative perspective*.

[j_jhsl-2019-0016_ref_039] Toorn M.C. van den, Pijnenburg W., Leuvensteijn J.A., van der Horst J.M. (1997). *Geschiedenis van de Nederlandse taal* [History of the Dutch language].

[j_jhsl-2019-0016_ref_040] Vezzosi Letizia (2000). The history of the genitive in Dutch: An evidence of the interference between language standardization and spontaneous drift. *Studia Germanica Posnaniensia*.

[j_jhsl-2019-0016_ref_041] Wal Marijke van der, van Bree Cor (2008). *Geschiedenis van het Nederlands* [History of Dutch]. *Houten: Spectrum*.

[j_jhsl-2019-0016_ref_042] Wal Marijke van der, Rutten Gijsbert (2013). *Touching the past: Studies in the historical sociolinguistics of ego-documents*.

[j_jhsl-2019-0016_ref_043] Weerman Fred, de Wit Petra (1999). The decline of the genitive in Dutch. *Linguistics*.

[j_jhsl-2019-0016_ref_044] Weerman Fred, Olson Mike, Cloutier Robert A. (2013). Synchronic variation and loss of the genitive case. Formal and informal language in a Dutch corpus of 17th-century Amsterdam texts. *Diachronica*.

[j_jhsl-2019-0016_ref_045] Weiland Pieter (1805). *Nederduitsche spraakkunst* [Dutch grammar].

[j_jhsl-2019-0016_ref_046] Wright Sue, Bernard Spolsky (2012). Language policy, the nation and nationalism. *The Cambridge handbook of language policy*.

[j_jhsl-2019-0016_ref_047] Yáñez-Bouza Nuria (2015). *Grammar, rhetoric and usage in English: Preposition placement 1500–1900*.

